# Perceived Barriers to the Use of Assisted Colonization for Climate Sensitive Species in the Hawaiian Islands

**DOI:** 10.1007/s00267-021-01491-w

**Published:** 2021-06-23

**Authors:** Shannon N. Rivera, Lucas Berio Fortini, Sheldon Plentovich, Melissa R. Price

**Affiliations:** 1 University of Hawai’i at Mānoa, 1910 East-West Road, Honolulu, HI 96822 USA; 2 U.S. Geological Survey, Pacific Island Ecosystems Research Center, 1845 Wasp Blvd, Bldg 176, Honolulu, HI 96818 USA; 3 U. S. Fish and Wildlife Service, Pacific Islands Coastal Program, 300 Ala Moana Blvd, Rm 3–122, Honolulu, HI 96850 USA

**Keywords:** Translocation, Climate change, Endangered species, Assisted migration, Conservation introduction

## Abstract

Conservation actions to safeguard climate change vulnerable species may not be utilized due to a variety of perceived barriers. Assisted colonization, the intentional movement and release of an organism outside its historical range, is one tool available for species predicted to lose habitat under future climate change scenarios, particularly for single island or single mountain range endemic species. Despite the existence of policies that allow for this action, to date, assisted colonization has rarely been utilized for species of conservation concern in the Hawaiian Islands. Given the potential for climate driven biodiversity loss, the Hawaiian Islands are a prime location for the consideration of adaptation strategies. We used first-person interviews with conservation decision makers, managers, and scientists who work with endangered species in the Hawaiian Islands to identify perceived barriers to the use of assisted colonization. We found that assisted colonization was often not considered or utilized due to a lack of expertize with translocations; ecological risk and uncertainty, economic constraints, concerns regarding policies and permitting, concerns with public perception, and institutional resistance. Therefore, conservation planners may benefit from decision tools that integrate risk and uncertainty into decision models, and compare potential outcomes among conservation actions under consideration, including assisted colonization. Within a decision framework that addresses concerns, all conservation actions for climate sensitive species, including assisted colonization, may be considered in a timely manner.

## Introduction

Changing climates may have significant ecological consequences for many species (Rosenzweig et al. 2008; Chen et al. 2011; Wiens 2016), increasing the need for a wider range of options in conservation management (Vitt et al. 2009; Bellard et al. 2012; Schwartz et al. 2012; Fortini et al. 2015). Some species will be able to respond to climatic shifts by migrating to suitable climates (Parmesan and Yohe 2003; Dawson et al. 2011). However, migration may not be an option for species with limited dispersal capabilities, specialized habitat requirements, decreasing habitat size, or other restrictions (Bellard et al. 2012; Taylor and Kumar 2016; Brito-Morales et al. 2018). Additionally, climatic shifts are happening at such an accelerated rate that local adaptation may be impossible for many species, particularly those with long generation times (Visser 2008; Williams et al. 2008; Quintero and Wiens 2013). Given these limitations, as many as two thirds of Earth’s extant terrestrial species may become extinct by the end of this century (Raven et al. 2011; Pimm et al. 2014; Cafaro 2015; Ceballos et al. 2015). Therefore, adaptation strategies are of critical importance to minimize extinction risk under a changing climate.

Assisted colonization is a translocation tool defined by the International Union for the Conservation of Nature (IUCN) as the “intentional movement and release of an organism outside of its historical range” (IUCN/SSC 2013). Assisted colonization may be one potentially suitable action for species at risk of extinction due to shifts in habitat caused by climate change (Vitt et al. 2009; Schwartz et al. 2012). Assisted colonization results in range expansions of single island and single location populations (Fischer and Lindenmayer 2000; McLachlan et al 2007; Freifeld et al. 2016) and minimizes the likelihood that a single catastrophic event will result in species extinction. Given concerns regarding a loss of habitat for climate sensitive species, assisted colonization could be considered alongside more commonly used conservation actions to achieve species recovery goals in a changing climate. However, it has rarely been utilized, or even considered, as a strategy to safeguard climate sensitive species.

Globally, island endemic species are some of the most affected by risks associated with climate change (Raxworthy et al. 2008; Sekercioglu et al. 2008; Freeman and Freeman 2014; Kougioumoutzis et al. 2020). The suite of challenges facing island endemic species, including invasive predators and competitors, habitat degradation, and the loss of mutually beneficial species, alongside economic and social challenges, lead to a complex decision environment (Price and Toonen 2017). In the Hawaiian Islands, projected declines of multiple, single-island endemic species have managers seeking long term options for the persistence and recovery of those species (Fortini et al. 2015, Paxton et al. 2018). For example, most remaining Hawaiian forest birds are constrained to higher elevations that are relatively cool and where avian malaria and its mosquito vector generally are absent (van Riper et al. 1986; Samuel et al. 2011). As temperatures increase, mosquitos expand their range, and many Hawaiian forest birds are projected to lose most of their habitat under future conditions (Fortini et al. 2015). Hawaiian plants are also vulnerable, particularly as their dispersal may be limited and they may not be able to establish populations in new locations with suitable conditions. By the year 2100, the distributions of more than 100 native plant species are projected to be disconnected from their current distributions (Fortini et al. 2013). Because the natural colonization of these species into new climate compatible areas is hindered by threats such as the predation of seeds and fruits by invasive animals and the expansion of invasive competitors (Mack et al. 2000; Courchamp et al. 2003), innovative management strategies have been increasingly discussed within the management community. A successful trial planting of *Cyanea superba*, an Oʻahu endemic plant, at higher elevations highlighted the possibility that some species may already be under stress from the documented multi-decadal drying trend across the state (Bassiouni and Oki 2013, Frazier and Giambelluca 2016). Endemic invertebrates, such as Hawaiian tree snails, are also vulnerable due to limited mobility, habitat loss, and the expansion of invasive predators, all of which compound physiological susceptibility to drought associated with climate change (Price et al. 2015; Price et al. 2021). Therefore, multiple taxonomic groups may benefit from assisted colonization.

Given the high climate vulnerability of multiple diverse taxa and the low level of adoption of assisted colonization as a conservation tool in Hawaiʻi, the archipelago offered an ideal setting to examine the major impediments to decision-making in regard to assisted colonization. We aimed to identify perceived barriers to the use of assisted colonization in the Hawaiian Islands by conducting first-person interviews with conservation decision makers, managers, and scientists. We then examined potential ways to alleviate impediments to decision making and support effective management of climate sensitive species.

## Methods

We identified participants based on their positions in state, federal, private, or nonprofit organizations with responsibilities for threatened and endangered species management. We used stratified snowball sampling, which allowed initial participants to recommend other potential participants with relevant experience; contacting and interviewing suggested individuals was left to the discretion of the researchers. Our participants represented a cross section of fields, taxonomic expertize, geographic locations, professional affiliations, and professional positions. We selected participants based on reputation, professional achievement, and familiarity with translocation management strategies. Collectively, participants came from national and international backgrounds, each with 10 to over 20 years of experience. Following the theoretical saturation model from Glaser and Strauss (1967), we ended selection of additional participants when the preceding interviews yielded no additional data. Our aim was not to obtain a statistically representative sample but to gain insights into the subject (Trost 1986). We reached saturation at 22 participants. The University of Hawai’i at Mānoa’s Institutional Review Board granted ethical approval to carry out the study; Protocol ID: 2016-30294A. We provided information sheets to each participant via email or in person to inform them of the study and to gain formal consent for their involvement.

### First-person Interviews

We designed a semi structured interview protocol and interviewed 22 people from 2016 through 2021. To ensure confidentiality, we coded participants as P1 through P22. The rigor of qualitative inquiry is established through trustworthiness (Lincoln and Guba 1985). Therefore, we offered member checks of transcripts to participants to ensure accuracy after the interviews were conducted. We conducted interviews in person or via telephone and recorded them with an audio recorder and handwritten notes. We deleted audio recordings after transcription and secured transcripts in a password protected file to ensure confidentiality.

We asked participants five guiding questions. (1) Describe the barriers you face in the process of implementing assisted colonization. (2) Are there any cases that you are familiar with in which assisted colonization could be considered? (3) What are your top concerns when considering the use of assisted colonization? (4) Are there any policies or procedures that would ease your use of assisted colonization? If yes, please explain what would make implementing assisted colonization easier. (5) Based on your expertize, where has this management option failed or succeeded, and where has it not been considered? To further clarify responses, researchers asked follow up questions, which differed among interviews. The question sequence and wording were adjusted at each interview to maintain a natural narrative.

### Analysis

We manually analyzed interviews with qualitative content analysis in which the key issues were grouped into emergent topics. Content analysis classifies written or oral materials into categories of similar meanings (Moretti et al. 2011). Qualitative content analysis is used for “subjective interpretation of the content of text data through the systematic classification process of coding and identifying themes or patterns” (Hsieh and Shannon 2005, p. 1278). First, we deductively defined codes based on empirical data and coded relevant statements from interviews with keywords deduced from early interviews. Second, we identified new topics with inductive coding. We entered themed statements and keywords into a database built with Microsoft Excel. A deductive inductive strategy allows for flexibility in analyzing the qualitative material. Codes were manually grouped into emergent themes by two researchers to check for consistency, and the finalized analyses were discussed and clarified amongst all authors to guard against biases. After coding, a rich description of perceptions relevant to perceived barriers to the use of assisted colonization was generated.

## Results

The perceived barriers to the use of assisted colonization identified by the 22 participants fell within nine categories (Table 1): economic constraints (21 participants), ecological risk and uncertainty (21 participants), lack of expertize (21 participants), institutional resistance (18 participants), policy and permitting (16 participants), lack of species data (16 participants), time commitments (6 participants), public perceptions (*n* = 6), and lack of capacity (*n* = 5). Below, we illustrate our findings with quotations from participants.

**Table 1:** Summarized perceived barriers to the consideration and use of assisted colonization

	P1	P2	P4	P6	P9	P11	P14	P16	P5	P7	P8	P12	P15	P18	P21	P3	P10	P17	P19	P20	P13	P22
	Fed Govt	Fed Govt	Fed Govt	Fed Govt	Fed Govt	Fed Govt	Fed Govt	Fed Govt	State Govt	State Govt	State Govt	State Govt	State Govt	State Govt	State Govt	NGO	NGO	NGO	NGO	NGO	Private	Private
Ecological risk & uncertainty	X	X	X	X	X	X	X	X	X	X	X	X	X	X	X	X		X	X	X	X	X
Lack of species data	–	–	–	X	X	X	X	X	X	–	X	X	X	X			X	X	X	X	X	X
Lack of expertize	X	X	X	X	X	X	X	X	X	X	X	X	X	X	X	X	X		X	X	X	X
Lack of capacity	–	–	X	–	X	–	–	–	–	–	–	X	–	–							X	X
Economic constraints	X	X	X	X	X	X	–	X	X	X	X	X	X	X		X	X	X	X	X	X	X
Policy & permitting	–	X	X	–	–	X	X	X	X	X	X	–	X	X	X		X	X	X	X		X
Time commitments	X	X	–	–	–	X	–	–	–	–	–	–	–	X					X			X
Public perceptions	–	–	–	–	–	–	X	–	–	–	–	–	–	–	X		X	X		X		X
Institutional resistance	X	X	X	X	X	–	X	X	X	X	X	X	–	X	X	X	X		X		X	X

Participants are labeled P1-P22 to ensure confidentiality and are grouped according to their professional affiliation: federal government (Fed Govt), State Government (State Govt), nongovernmental organization (NGO), private. ‘X’ indicates that the participant described barriers that fall within the nine defined categories

### Economic Constraints

Economic constraints and funding uncertainty inhibited the consideration of assisted colonization. Some of the costs associated with assisted colonization were completing a translocation plan, obtaining permits, implementing the plan and conducting monitoring, captive propagation, animal husbandry, and habitat restoration. Economic constraints had the potential to mitigate the challenges posed by other barriers, as captured by P19: “We are not translocating things we need to because we don’t have the data and yet we don’t want to spend the money [to practice] our translocation techniques on [a species] that’s common. So probably we should have put some money into those common species. Funding is certainly a huge obstacle. Another huge obstacle is lack of knowledge. And another huge obstacle is permitting. Funding can help with all those things”.

Moving individuals from one island to another might reduce funding for the source island if the translocated population acted as an umbrella species that garners conservation funding that also benefits other conservation objectives. P5 explained, “if you remove a bird [species] from a particular island then the funding and focus may be taken from conservation efforts on that island”.

Substantial operating costs were associated with planning and implementing any type of translocation action. All participants noted that budgets were limited for any conservation management option and were not restricted to assisted colonization. P3 made the point that “conservation management is not supposed to take money or budget into consideration”. Similarly, P15 stated, “funding’s always going to be an issue no matter what you choose, especially conservation in Hawaiʻi”.

Two participants revealed that although funding is always a concern, assisted colonization could be less expensive than other conservation strategies for certain species. P21 commented, “[assisted colonization] would actually be cheaper in some instances because Oahu’s upper elevation habitats are really remote and hard to get to, whereas other islands have access points to remote areas that are [ideal] for constructing predator proof fencing…I would argue that in our situation, moving things to other islands to help recover them might actually be a cheaper option than having to fly materials and fly staff constantly to these really remote areas”.

### Ecological Risk and Uncertainty

Participants recognized that models of the impacts of climate change exist for many species of concern in Hawaiʻi, such as forest birds and plants, but not for most invertebrates. Even for species for which impact models exist, we found that the inherent uncertainties in modeled projections, or even a lack of comfort with understanding complex models or ecology, may result in resistance to planning or acting on the basis of model outputs. P10 explained how this could differ depending on the species: “there are some [species] that are just inherently less risky to try [assisted colonization] with because you don’t have as much to lose if a bunch of them die as opposed to a species that is really slow breeding and has very complex social interactions”.

P20 shared an example of a concern about increases in ecological uncertainty when moving a species outside of its historical range: “If you’re moving a bird to part of its indigenous range where it just hasn’t been for 30 years, we aren’t concerned about that. But when you start talking about moving things like kiwikiu [*Pseudonestor xanthophrys*] to Big Island [Hawaiʻi Island], now you have a bird that has never been there. [It’s] a whole entire new ecosystem, so you don’t have any idea of how it’s going to interact”.

Other ecological uncertainties that were mentioned included potential risks to translocated individuals (e.g., direct mortality, genetic bottlenecks, inbreeding depression, hybridization) and potential risks and impacts to the recipient ecosystem (e.g., disease transmission, the potential for introduced species to become invasive). P18 highlighted one concern associated with disease transmission: “There would be other concerns such as ʻwhat if the reason [the translocated species] is not doing well in its native range… is due to some sort of heritable pathogen and then we move it. We don’t know much about the microbiome associated with insects or their pathogens in general… we are just kind of flying blind”.

### Lack of Expertize

‘Lack of confidence’ and ‘unfamiliarity’ appeared to be barriers to the consideration of assisted colonization. P10 provided insight into how lack of experience may create a perceived barrier: “Ultimately everyone within conservation wants the same thing. They want the animals to get to where they can be safe successfully… Maybe it’s a difference in the level of confidence in the techniques. Just a lack of expertize or not feeling comfortable in doing it. I think just with any person, any manager, you want to stick to what you’re good at, and there’s not really a confidence in knowing how to do this yet”.

Twenty one participants noted that all other concerns regarding the use of assisted colonization, including some aspects of ecological risk and uncertainty, could be ameliorated through gaining experience with this management tool. P14 suggested, “[A local experienced agency] should create guidance on assisted colonization. That would alleviate some of the institutional resistance”. Fifteen participants expressed interest or support for increasing knowledge of assisted colonization by using less vulnerable species to set a precedent. P10 shared an example: “I am personally interested in the idea of assisted colonization of ʻamakihi (*Loxops virens*) [to Lanaʻi]. There was a subspecies [on Lanaʻi], they were extirpated, but there are some low elevation birds that are doing pretty well on other islands [such as ʻamakihi] that could be a potential source. For government agencies, these birds may not be a priority for them because they’re not super threatened, but it would be returning some functionality to a native ecosystem and also you might learn some techniques or tricks on something that isn’t as endangered before you have to try it on our critically endangered birds with 80 or 100 individuals left”.

### Institutional Resistance

Risk aversion by management authorities, or institutional resistance, was identified by 18 participants as hindering the consideration of assisted colonization. Resistance was associated with unfamiliarity with the strategy, unwillingness to implement high risk approaches, and fear of career repercussions. P19 disclosed, “people lose their jobs if a bird is killed, if an individual dies on their watch. So sometimes that’s the problem. No one loses their job over a species going extinct as far as I can tell, but people do lose their job over killing one bird, or they have consequences. So that’s really hard… That’s one thing that we need to look into is how we can protect people who take well considered and well informed risks to protect species”.

Emotional inhibition tied to negative past experiences also created resistance to the consideration of assisted colonization. P10 described the following experiences: “At a couple of meetings where, I wouldn’t call it knock-down-drag-out fights, but there were people in tears because of how strongly they disagreed with each other about whether assisted colonization was the only option left for the species…Failures [have] caused a fair amount of hesitancy within the conservation community. Some people are unwilling to try it, regardless of the amount of planning”.

Conflicting ideologies also influenced institutional resistance. P22 said, “I don’t think there’s a whole lot of funding available for something that is as new as [assisted colonization] and we’ve just gone through four years of denial that climate change is actually happening, so that kind of pushed us backward… in regard to funding, but also just general support for doing these types of things”.

Although assisted colonization has been viewed as controversial (Fazey and Fischer 2009; McLachlan et al. 2007; Ricciardi and Simberloff 2009), the alleviation of resistance was a central theme during interviews. Seven participants commented that in recent years, hesitation about assisted colonization has eased and perceptions have changed. Participants felt that these changes were associated with people becoming more comfortable with the strategy after years of discussion, the failure of traditional strategies, and as the increased risk of extinction. P19 commented, “I’ve definitely seen a change and I’ve been here 10 years. That culture is changing because [some managers] realized that their inaction, their unwillingness to accept risk, led to extinction”. Similarly, P20 said, “Everybody wants to conserve the species, nobody wants anything to go extinct. The disagreements all tend to be a different value of the risks and likelihood of success, of different tactics and different perceptions, different experiences. [Assisted Colonization] is not a silver bullet that’s going to solve the conservation issues. We are in desperate times for multiple species and multiple locations in Hawaiʻi, so all the options need to be considered. Everyone understands that”.

### Policies and Permitting

Sixteen participants identified policy and permitting as barriers to the consideration of assisted colonization, recognizing that maneuvering through translocation policy and the permitting process can be restrictive and difficult. P21 shared, “I don’t think the policy is insurmountable, but I do think [assisted colonization] would be significantly harder and would require more consultation”. Participants observed that assisted colonization is a potential option under both federal and state law as it is allowed under the Endangered Species Act (ESA) under Section 10(j) and the experimental population provision (ESA, 16 U.S.C. 1539(j)(1)) and under Hawaiʻi Revised Statute (HRS) 195D-29 ‘release or establishment of endangered or threatened species outside its current range’ (HRS, 195D-29 L 1997, c 380, pt of §2) with approval from the state’s Endangered Species Recovery Committee. Seven participants noted the potential for misinterpretation of policy. P16 explained that although it may be legal, individual agreements could be prohibitive: “our cooperative agreement with the Fish and Wildlife Service, which hasn’t been revisited since the mid 1980s, explicitly states that we’re not allowed to… move endangered species outside of their known historical range. So technically we are still not allowed to do that and wouldn’t be unless that agreement changed”.

P12 highlighted the complexity of management by multiple jurisdictions: “If it was federal land and we wanted to collect a listed, endangered species then there is a whole other regulatory process, but the federal government doesn’t own much land in Hawaiʻi, so there’s not a federal nexus for them to infringe on these things for plants. But that would be different if you had an animal. Plants are considered part of the landscape and the animals are considered a federal jurisdiction. The Fish and Wildlife Service doesn’t assume responsibility for any plants on state property”.

### Lack of Species Data

Sixteen participants raised concerns about gaps in knowledge of candidate species, particularly cryptic or rare species for which there may be little information on behavior or habitat, such as foraging, breeding habitat, and historical ranges. One participant noted that many invertebrates haven’t been described by science, and therefore understanding of their conservation status or management needs has not begun. P13 highlighted, “it is difficult to establish the historical range for species that are rare, cryptic, and are not present in the fossil record”. P19 shared an example regarding a Kauaʻi endemic forest bird, “One of our major barriers to translocating ʻakekeʻe [*Loxops caeruleirostris]* or doing anything with akekeʻe, even before a habitat suitability study, is that we actually know next to nothing about the species”.

Lack of species data was closely associated with ecological risks and uncertainties. P12 commented, “we have few years of experience with some species. Especially when species have one or few or no populations remaining [in the wild], it is hard to know preferred habitat requirements as what we see may not be optimal”. Similarly, P16 noted, “there’s a likelihood that where remaining endangered plants occur in the landscape over the last 60 years are not necessarily representative of where they were across the landscape as a whole. Those [historical] maps are a hypothesis of where those plants could have occurred…it could be misleading, and it may not be appropriate with climate change. They give us a picture of the niche of that species, but they could be in the worst possible habitat for that species, which is what I suspect for some [Hawaiian] plants”.

### Time Commitments

Six participants indicated that the length of time necessary to plan and implement assisted colonization is a barrier. Discussing previous management decisions for birds, P1 stated, “the option of assisted colonization was taken off the table early on, at least as an immediate option because it would not be immediate; assisted colonization is a detailed process”. Translocation is a long term commitment, and future funding is often uncertain. P18 shared an example: “So often, our grants are only two or three year grants, so by the time we have the funding to start working on something, we can’t afford the time to devote to that”.

P19 shared an example of a management decision from several years ago: “We didn’t really have any other strategy to look into because the time horizon for planning a translocation is lengthy and we wanted to do something that we could do more quickly because there’s so much more paperwork for a translocation… maybe we should have investigated translocation more seriously early on because if it works, it’s going have a bigger payoff, but it’s just super risky”.

The low population sizes of many Hawaiian species also relate to time constraints and may not be possible to overcome even with increased institutional support, data, or funding. P19 acknowledged that “slow but surely, we can chip away at gathering the ecological data. Slowly but surely, we are getting funding because people are concerned. But things move slowly and sometimes species don’t have time for us to move slowly. So that’s another problem”.

### Public Perceptions

Negative public perceptions, or community resistance, were perceived as a barrier to the consideration of moving species outside of their historical range. This barrier was a greater consideration for well known, popular, or culturally important species. For example, P15 said, “[Some birds] certainly get a lot more spotlight. That means we might not be as willing to take risks as we might with, say, a plant or an invertebrate species. If [you] try a translocation and it fails, with a bird species there might be more public backlash than if that happened with another species group. So that is a concern”.

Interviews suggested community aversion to moving species from their native areas, and potential community resistance to receiving new species. P17 commented, “I think we’re very much aware of the need for community support. We need to justify why we’re spending the money that we do on conservation because it is taxpayer dollars a lot of the time. Also, to have coexistence, you need to have people be excited and accepting of species in their backyard. For [some species] that is not always the case”.

Community resistance was not perceived as a concern for all species. Rather, the lack of community support and knowledge of certain species impacted conservation support. P19 said, “[For] very iconic species that people instantly recognize, [the public] probably has more attachment to it. The little species I work on have been restricted to the darkest reaches of the island for so long, that very few residents care what they are. Unfortunately, people don’t even know our [species] and that’s actually a barrier to conservation”.

### Lack of Capacity

Five participants identified lack of capacity as a barrier to the consideration and use of assisted colonization. Capacity concerns were associated with planning and implementation, data collection and dissemination, knowledge sharing, and policy and permitting. P9 shared an example where capacity was the greatest barrier to the use of assisted colonization for a particular species’ recovery plan: “we have a project where we want to move a species outside of its original range. It’s something that we probably should have done four years ago, and we still don’t even really have it started. For us…our obstacle is not having someone to do the planning work for it. We don’t have anyone who has the time… We haven’t even gotten to funding or anything like that yet, but it’s a lack of capacity”.

Lack of capacity was associated with a lack of data and limited dissemination of information that could increase expertize and mitigate ecological uncertainty. P12 shared several examples of implementing assisted colonization but lacking capacity to move beyond management and practice: “It’s really hard for me, as a manager, to have the time to publish. Maybe we don’t take enough robust data; we might monitor the [species] populations once a year, so we can’t determine specifically why exactly 10% died from slugs and 5% died from drought, or something like that. We don’t know those types of answers”. P7 noted that permitting constraints were also tied to limited capacity, saying, “more capacity is needed at [permitting agency] to expedite permits for threatened and endangered species”.

## Discussion

**Fig. 1:**
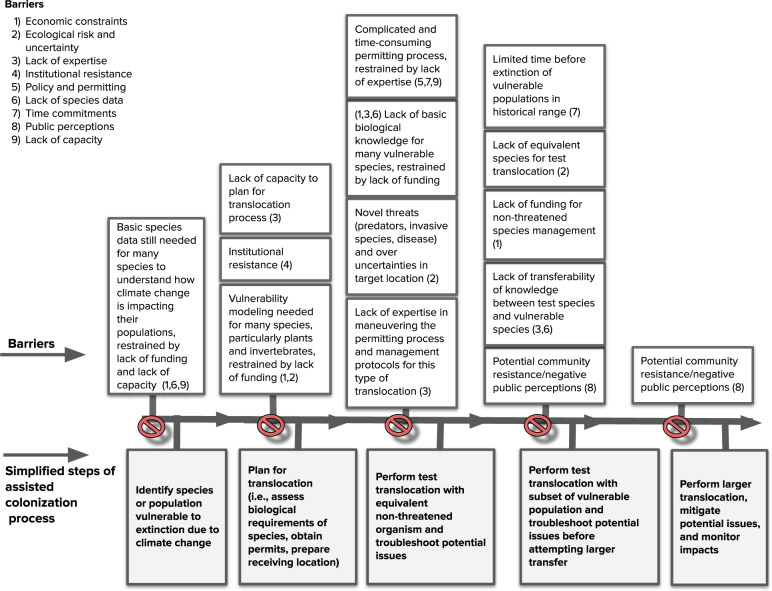
Perceived barriers identified from this study shown in relation to the different stages of a theoretical simplified assisted colonization process

We identified major impediments to making decisions about the use of assisted colonization in the Hawaiian Islands (Fig. 1) and factors influencing the persistence of these barriers. The perceived barriers are shaped by societal, governmental, and personal influences.

Funding concerns were a central theme during interviews, but economic considerations were often confounded with nearly all other perceived barriers. Participants established that there is a preference for strategies that produce visible, immediate results that can be demonstrated to funding agencies. The interests of funding bodies, in turn, were perceived to strongly influence management approaches. Institutional resistance and the lack of personal will to invest in controversial methods may also interact with funding decisions. Assisted colonization may be viewed as irrelevant when timelines for making decisions are largely shaped by annual funding cycles. Translocations require years of planning and long term monitoring. The lengthy process of attempting a translocation outside of the species’ historical range could be a deterrent (Wolok 2001). As mentioned by participants, regional politics may play a role. Assisted colonization may be perceived as giving up on previously identified habitat within the historical range, with potential loss of interest or funding for actions that benefit coexisting species.

The uncertainty of encountering adverse ecological interactions in the recipient ecosystem (e.g., competition between the introduced taxon and local threatened taxa) has been raised by others (McLachlan et al. 2007; Hoegh-Guldberg et al. 2008; Ricciardi and Simberloff 2009; Loss et al. 2011; Schwartz et al. 2009). The challenges associated with invasive species that managers encounter daily may increase the perceived risk of issues believed to be related, even if they are not logically connected (Mrkva et al. 2021).

Similarly, managers may feel more strongly about threats perceived to be directly impacting species at present, rather than those that may impact species in the future, and act accordingly (Chang and Pham 2012). As a result, although impact models have been built for all Hawaiian forest birds and 1100 species of Hawaiian plants (Fortini et al. 2013; Fortini et al. 2015), decision makers may be uncomfortable taking action on the basis of these models because of the complexity of interactions causing species declines in the Pacific. Furthermore, models for many species have only recently been incorporated into US Fish and Wildlife Service (USFWS) recovery plans. Through 2008, 87% of species recovery plans did not acknowledge climate change as a threat (Povilitis and Suckling 2010), but in the last decade, more plans have incorporated climate change (Fischman et al. 2014).

Managers prefer actions for which confidence in the outcomes is high (Andreoni and Sprenger 2012). Furthermore, the potential benefits of a given action for species recovery may not be measurable for several decades, and decision makers prefer actions for which responses can be measured immediately (Fajfar et al. 2012). Lack of expertize was identified as a hindrance to the consideration and especially the implementation of assisted colonization. Although perceptions were identified as changing, conservation professionals were identified by participants to prefer tested and familiar approaches. Individuals are more likely to take a familiar course of action than a novel action (Bekir and Doss 2020). Becoming more familiar with the idea of moving species outside of their historical range to prevent extinction could alleviate community resistance.

Time discounting (Abdellaoui et al. 2019) means that managers may place a higher priority on current threats, such as predation, than threats perceived to impact species in the future. Structured decision making processes, which frame uncertainty in the context of management objectives, species models, and risk tolerance (McLachlan et al. 2007; Ricciardi and Simberloff 2009; Loss et al. 2011; Schwartz et al. 2009) may provide a means of assessing the likelihood of success under various scenarios and accounting for heuristics such as time discounting. The combination of time discounting with the time commitments required and lengthy permitting processes may discourage unconventional conservation decisions such as assisted colonization. If population sizes continue to decline, it may not be feasible to reconsider translocation in the future.

State and federal policy and regulations in the United States may allow for assisted colonization. However, there is some potential for misinterpretation of policy. In 1984, the USFWS restricted relocation outside a species’ historical range unless “the primary habitat of the species has been unsuitably and irreversibly altered or destroyed” (50 C.F.R. §17.81(a)). The agency’s position was “that the relocation or transplantation of native listed species outside their historic range will not be authorized as a conservation measure” (49 FR 33890). Nevertheless, the USFWS will authorize release outside the current range if “release will further the conservation of such species” (ESA, 16 U.S.C. §1539(j)(1)). Managers may not consider relocation due to lack of familiarity or lack of personal or institutional will to navigate the regulatory system.

### Alleviating the Perceived Barriers

Despite successes, and the numerous threats to species around the world, assisted colonization is rarely considered during conservation planning processes (Fazey and Fischer 2009; Ricciardi and Simberloff 2009; Neff and Carroll 2016). Much of the apprehension surrounding the use of assisted colonization, and translocations in general, may be alleviated by experienced leaders who are proficient in translocation procedures including permitting, habitat preparation, husbandry, and cultural knowledge. Initial adopters familiar with this management option may then support others considering the strategy through both formal and informal networking and training (Freifeld et al. 2016).

Managers may delay consideration of assisted colonization while waiting for other tools to be developed if those tools are perceived as providing a higher chance of recovery. For example, as avian malaria spreads to higher elevations on Kauaʻi, managers may delay assisted colonization due to the ongoing effort to develop new mosquito control techniques (Harvey-Samuel et al. 2021).

Acknowledgment of concerns may allow for critical evaluation of perceived risks, particularly given the multiple threats facing species. The possibility of any additional pressure on endemic and endangered Maui and Hawaiʻi species was expressed by managers, as population sizes of many native species are likely declining (Fortini et al 2013; Fortini et al. 2015). However, evaluation of potential translocations of critically endangered single-island endemics from Kauaʻi to Maui or Hawaiʻi island indicates that there is minimal potential for competition and range overlap among candidates for translocation and resident native species (Mountainspring and Scott 1985; Scott et al. 1986, Fortini et al. 2017).

Another source of ecological uncertainty is causes of prior extinctions of related species. For example, ʻAkekeʻe (*Loxops caeruleirostris*) are likely to become extinct on Kaua’i in the near future as mosquito borne avian malaria reaches the highest elevations of the island (Fortini et al. 2015). This bird is closely related to the extinct Maui ʻĀkepa (*L. ochraceus*). On the one hand, the introduction of ʻAkekeʻe into Maui could be a rare opportunity to restore ecological function lost by the extinction of ʻĀkepa. On the other hand, without an understanding of the causes of extinction of Maui ʻĀkepa, the introduction may fail.

Given these levels of uncertainty, decision support tools may help determine a suite of actions with the highest probability of achieving long term recovery of endangered species (McDonald-Madden et al. 2010; Burbidge et al. 2011; Loss et al. 2011). For assisted colonization to be a viable strategy, regional decision frameworks may be developed that allow assisted colonization to be considered while population sizes are still sufficient. Population sizes of many species in the Hawaiian Islands are smaller than optimal for any type of translocation, even following captive propagation (Lenton et al. 2008; Vitt et al. 2010). With emerging recognition that the viability of assisted colonization needs to be evaluated on a species and system-specific basis, several strategies have been developed to aid decisions (McDonald-Madden et al. 2010; Burbidge et al. 2011; Loss et al. 2011). Furthermore, different decision tools may be necessary to address multiple objectives, risk, and uncertainty. Therefore, it is important to identify the major impediments to decision making.

Decision makers, managers, and researchers from regions with a high number of species that may benefit from assisted colonization may wish to develop communities of practice around translocations in general and assisted colonization in particular. Creating a community of practice may advance the consideration of novel management tools, particularly because effective conservation often requires collaborative relationships. Many regions may also benefit from local planning. One such planning step is identifying candidates for assisted colonization before population sizes fall below those that are recommended to maximize the likelihood of successful translocations. An effort to document translocation successes and failures may be particularly useful. With a network of practitioners, documentation of best practices and recognition that mitigating negative perceptions will mitigate perceived barriers may enable assisted colonization to be considered in a timely manner for vulnerable species that may become extinct due to climate change.
